# Booster dose of mRNA vaccine augments waning T cell and antibody responses against SARS-CoV-2

**DOI:** 10.3389/fimmu.2022.1012526

**Published:** 2022-10-12

**Authors:** Feyza Gül Özbay Kurt, Alisa Lepper, Catharina Gerhards, Mathis Roemer, Samantha Lasser, Ihor Arkhypov, Rebekka Bitsch, Peter Bugert, Peter Altevogt, Cécile Gouttefangeas, Michael Neumaier, Jochen Utikal, Viktor Umansky

**Affiliations:** ^1^ Skin Cancer Unit, German Cancer Research Center (DKFZ), Heidelberg, Germany; ^2^ Department of Dermatology, Venereology and Allergology, University Medical Center Mannheim, Ruprecht-Karl University of Heidelberg, Mannheim, Germany; ^3^ DKFZ-Hector Cancer Institute, University Medical Center Mannheim, Mannheim, Germany; ^4^ Mannheim Institute for Innate Immunoscience (MI3), Medical Faculty Mannheim, University of Heidelberg, Mannheim, Germany; ^5^ Institute for Clinical Chemistry, Medical Faculty Mannheim, University of Heidelberg, Mannheim, Germany; ^6^ German Red Cross Blood Service Baden-Württemberg – Hessen, Institute of Transfusion Medicine and Immunology, Medical Faculty Mannheim, Heidelberg University, Mannheim, Germany; ^7^ Department of Immunology, Institute for Cell Biology, University of Tübingen, Tübingen, Germany

**Keywords:** SARS-CoV-2, mRNA vaccine, vector vaccine, booster dose, T cell response, antibodies

## Abstract

A gradual decay in humoral and cellular immune responses over time upon SAR1S-CoV-2 vaccination may cause a lack of protective immunity. We conducted a longitudinal analysis of antibodies, T cells, and monocytes in 25 participants vaccinated with mRNA or ChAdOx1-S up to 12 weeks after the 3^rd^ (booster) dose with mRNA vaccine. We observed a substantial increase in antibodies and CD8 T cells specific for the spike protein of SARS-CoV-2 after vaccination. Moreover, vaccination induced activated T cells expressing CD69, CD137 and producing IFN-γ and TNF-α. Virus-specific CD8 T cells showed predominantly memory phenotype. Although the level of antibodies and frequency of virus-specific T cells reduced 4-6 months after the 2^nd^ dose, they were augmented after the 3^rd^ dose followed by a decrease later. Importantly, T cells generated after the 3^rd^ vaccination were also reactive against Omicron variant, indicated by a similar level of IFN-γ production after stimulation with Omicron peptides. Breakthrough infection in participants vaccinated with two doses induced more SARS-CoV-2-specific T cells than the booster vaccination. We found an upregulation of PD-L1 expression on monocytes but no accumulation of myeloid cells with MDSC-like immunosuppressive phenotype after the vaccination. Our results indicate that the 3^rd^ vaccination fosters antibody and T cell immune response independently from vaccine type used for the first two injections. However, such immune response is attenuated over time, suggesting thereby the need for further vaccinations.

## Introduction

In December 2019, the novel beta coronavirus, severe acute respiratory syndrome coronavirus 2 (SARS-CoV-2), has emerged, infected humans, and led to coronavirus disease 2019 (COVID-19) (1). Several effective vaccines were developed and applied in various countries to fight the pandemic disease.

The structure of the SARS-CoV-2 virion is comprised of the nucleocapsid (N), membrane (M), envelope (E), and spike (S) proteins (2, 3). S protein consists of two subunits, S1 and S2. While S1 contains the receptor binding domain (RBD), which has a crucial function in the virus entry into cells through interaction with human angiotensin converting enzyme-2 (ACE2) receptor, S2 has other basic elements to mediate membrane fusion. Moreover, S protein plays a crucial role in the induction of neutralizing antibodies and T-cell responses (2, 3). Most current authorized vaccines rely on the translation of viral S messenger RNA (mRNA) to protein (4). Two main technologies were used to manufacture SARS-CoV-2 vaccines: mRNA-based vaccines and viral vector-based vaccines. RNA-based vaccines like BNT162b2 from BioNTech and mRNA-1273 from Moderna use mRNA to deliver genetic information to produce S glycoprotein antigen (5, 6). The viral vector-based vaccines such as ChAdOx1-S (ChAd) from AstraZeneca and Ad26.COV.2.S from Johnson and Johnson employs non-replicating adenovirus as a vehicle to deliver the genetic code of S antigen (7). Results from earlier clinical trials demonstrated that both vaccines could generate a significant amount of neutralizing antibodies and virus-specific T cells, leading to the protection against COVID-19 (6, 8–10).

A coordinated response between the humoral and cellular immune system is required in host defense against SARS-CoV-2 (11). Most protective antibody responses are dependent on the help of CD4 T cells (12). Induction of virus-specific CD8 T cells was shown to correlate with the viral clearance and to interact with the innate immune responses in disease control (13). Moreover, the generation of effective T cell memory is critical to promote a long-term protective immunity to SARS-CoV-2 (14). Several reports have shown that both mRNA and vector vaccines elicit virus-specific memory T cell responses (15–17). Long-term monitoring of T cell responses is important to understand the effectiveness of protection against SARS-CoV-2.

Emergence of different variants has generated concern about the effectiveness of immunity after vaccination (18, 19). In November 2021, the Omicron variant (B.1.1.529) has rapidly become a dominant strain in most countries (20). Carrying a large number of mutations in its spike protein, this variant has a stronger binding capacity to human ACE2 protein (21) and an increased transmissibility (22) than the original strain. A number of reports demonstrated that 3 doses of vaccination generate higher responses against Omicron than 2 doses (23–25). Furthermore, T cells could play a critical role in preventing severe COVID-19 caused by Omicron due to their ability to provide long-lasting immunity and recognize the virus through different sites (26).

Numerous viruses were shown to decrease the frequency of myeloid antigen presenting cells and expand myeloid-derived suppressor cells (MDSC), a heterogeneous population of myeloid cells inhibiting functions of T and NK cells (27), as an immune evasion strategy (28). It was reported that functionally active monocytic (M)-MDSC accumulated in COVID-19 patients (29). However, a detailed understanding of the alterations in myeloid cells during vaccination is lacking.

In the present study, we characterized T cell and antibody responses as well as changes in myeloid cells, starting from the baseline up to 3 months after the 3^rd^ dose (booster) vaccination. Moreover, the level of antibodies and virus-specific T cells were measured in persons with breakthrough infections after two doses of vaccination. We found that the 3^rd^ dose strongly enhanced both antibody and T cell responses, which decreased 6 months after the 2^nd^ dose. Furthermore, we demonstrated the accumulation and activation of virus-specific memory CD8 T cells induced by vaccination. Importantly, after the 3^rd^ dose, T cells showed reactivity against the Omicron variant. Hence, our findings highlight the significance of booster vaccination in strengthening waning T cell and antibody-mediated immunity.

## Methods

### Participant recruitment and sample collection

**Figure 1:**
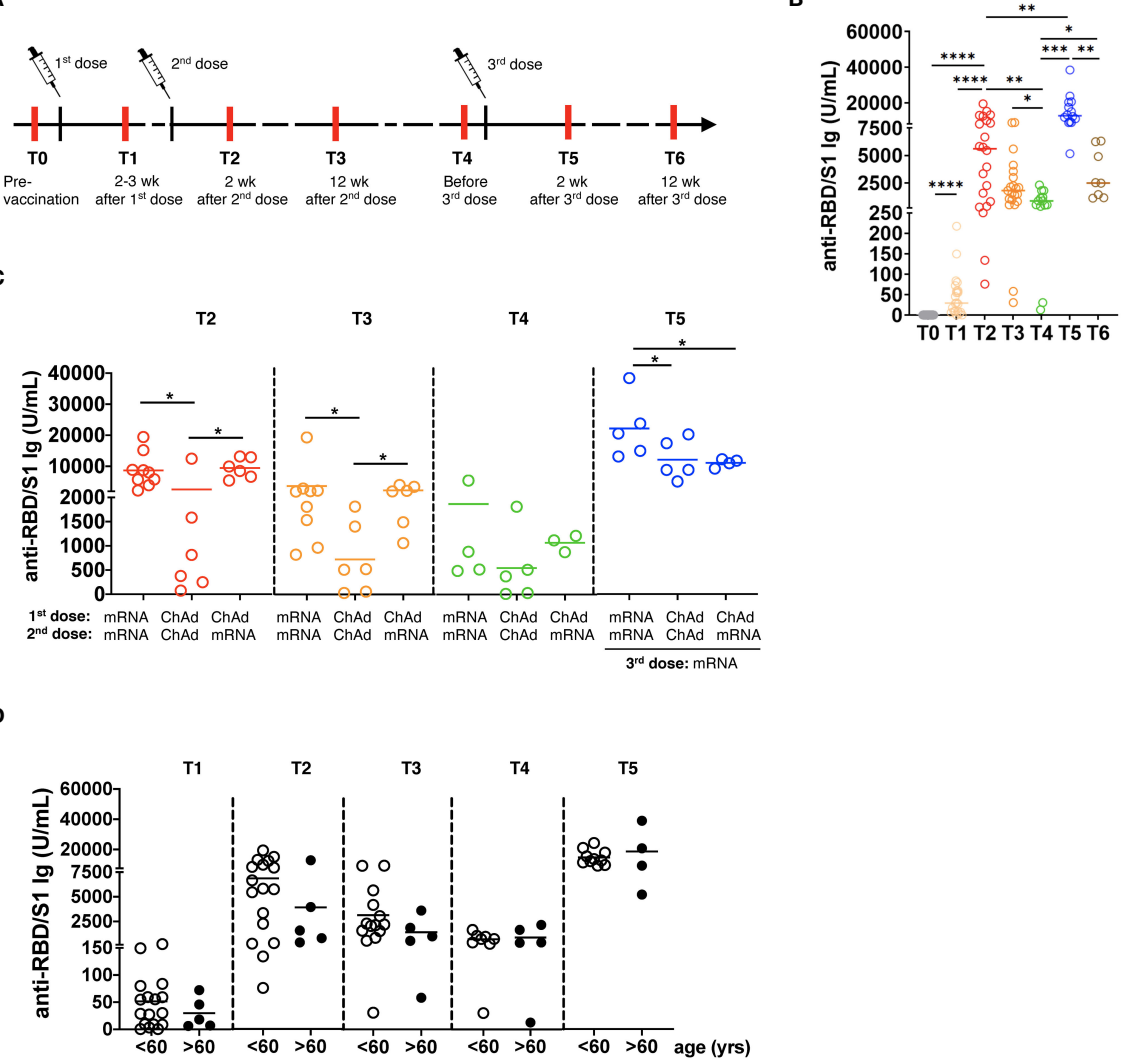
Generation of SARS-CoV-2 specific antibodies following vaccination. **(A)** The study design is presented. Red lines show the time of blood draw. **(B)** Total anti-spike RBD/S1 immunoglobulins (Ig) were measured in the serum of participants by the quantitative electrochemiluminescence immunoassay (ECLIA) and presented as U/mL (n=21). **(C)** Anti-RBD/S1 Ig levels in the serum of participants vaccinated two times with mRNA (mRNA/mRNA, n=9), two times with ChAd (ChAd/ChAd, n=6) or with ChAd/mRNA (n=6) are expressed as U/mL. **(D)** Anti-RBD/S1 Ig are measured in participants of >60 years (n=5) or <60 years (n=16) old and expressed as U/mL. wk: weeks; yrs: years; *P<0.05, **P<0.01, ***P<0.001, ****P<0.0001.

Participants were enrolled from February 2021 to April 2022 at the University Medical Center Mannheim, Germany (**Supplementary Table 1**). Written informed consents were obtained from all the participants prior to the study that was approved by the local Ethics Committee (2010-318N-MA and 2020-556N). Peripheral blood was collected at the following time points: pre-vaccination (T0), 2-3 weeks after the 1^st^ vaccination (T1), two weeks (T2) and 12 weeks after the 2^nd^ vaccination (T3), before the 3^rd^ vaccination (4-6 months after the 2^nd^ vaccination; T4), two weeks (T5) and 12 weeks after the 3^rd^ vaccination (T6; **Figure 1A**). In addition, we collected data regarding local and systemic solicited adverse events after vaccination. In the grading of adverse events, we used an FDA Toxicity Grading Scale (30). For those participants who got infected with SARS-CoV-2, blood samples were collected three and twelve weeks after the disease recovery confirmed by the PCR test.

HLA typing was performed according to the standard protocol. HLA-A alleles of participants are shown in **Supplementary Table 2**. Heparinized blood samples were subjected to the density gradient using Biocoll (Biochrom). Peripheral blood mononuclear cells (PBMC) were isolated *via* density gradient centrifugation using Biocoll (Biochrom) and cryopreserved in X-VIVO medium (Lonza) supplemented with 30% fetal bovine serum (FBS, Pan Biotech) and 10% DMSO in liquid nitrogen. Serum samples were collected and stored at -80°C.

### Anti-SARS-CoV-2 antibody detection

Analysis of total anti-spike RBD/S1 Ig was performed in serum by the quantitative electrochemiluminescence immunoassay (ECLIA) Elecsys^®^ Anti-SARS-CoV-2 S (Roche, Germany) assay approved by FDA and CE. After internal verification according to ISO 15189, the analysis was conducted according to the manufacturer’s instructions. The results were presented in U/mL and considered positive if ≥0.8 U/mL. SARS-CoV-2 anti-N Ig were detected by the qualitative Elecsys^®^ anti-SARS-CoV-2 assay (Roche, Germany) approved by FDA and CE. Values with a cut-off index (COI) ≥ 1.0 were considered positive.

### Flow cytometry

For MHC I dextramer staining, we chose two epitopes specific for each HLA-A subtype (**Supplementary Table 3**) from the SARS-CoV-2 derived epitopes with the strongest predicted affinity to MHC class I molecules defined by Immudex (Copenhagen, Denmark). PBMC (2x10^6^) were thawed, washed, and rested in X-VIVO medium containing 10% FBS for 1.5 h followed by the staining with pooled dextramers for each HLA-A subtype according to the Immudex protocol. Dead cells were excluded using Fixable viability stain-BV510 (BD Biosciences). The monoclonal antibodies (mAbs) for extracellular markers are listed in **Supplementary Table 3**.

The production of ROS and NO by myeloid cells was detected using CellROX™ Deep Red reagent and DAF-FM diacetate (Thermo Fisher), respectively.

For intracellular staining of IFN-γ and TNF-α, PBMC (2x10^6^) were rested overnight in X-VIVO medium containing 10% FBS and cells (2x10^6^) were stimulated in X-VIVO medium containing 5% human serum with pooled 0.6 nmol (1 μg) of overlapping 15mer peptides spanning the immunodominant sequence domains of spike SARS-CoV-2 protein (PepTivator SARS-CoV-2 Prot_S, Miltenyi Biotech) or CMV pp65 peptide pool (Miltenyi Biotec) for 6 h at 37°C and 5% CO_2_ in the presence of anti-CD28 monoclonal mAbs (1 μg/mL; Beckman Coulter). PBMC cultured in the medium with 2% sterile water or stimulated with phorbol myristate acetate (PMA; 50 ng/mL) and ionomycin (1μM) (Sigma Aldrich) were used as a negative control and positive control, respectively. In some experiments, cells were stimulated with a peptide pool spanning mutated regions in the BA.1 variant of Omicron variant (PepTivator SARS-CoV-2 Prot_S B.1.1.529/BA.1 Mutation pool, Miltenyi Biotec) or the same peptide pool with the original Wuhan strain sequence (PepTivator SARS-CoV-2 Prot_S B B.1.1.529/BA.1 WT Reference pool, Miltenyi Biotec) used as a control. After 2 h of incubation, GolgiStop (BD Biosciences) was added, and cells were further incubated for 4 h followed by the fixation and permeabilization with Cytofix/Cytoperm fixation/permabilization solution kit (BD Biosciences). Acquisition was performed using FACSLyric™ (BD Biosciences), and data were analyzed using FlowJo v10 software (BD Biosciences).

### Statistical analysis

Statistical analysis was performed using GraphPad Prism software version 8.3.1. Data distribution was determined by Shapiro-Wilks normality test. The Wilcoxon signed-rank test was used to compare paired samples. The Wilcoxon rank-sum test (Mann–Whitney *U* test) was used to compare unpaired samples and the Kruskal–Wallis *H* test was used to test for differences between multiple study groups. A value of p < 0.05 was considered statistically significant.

## Results

### Participant characteristics

Out of 25 recruited participants, 13 received homologous vaccination with mRNA vaccines BNT162b2 (n=12) and mRNA-1273 (n=1), 6 had homologous vaccination with ChAd and 6 were vaccinated with ChAd and BNT162b2 (**Supplementary Table 1**). Furthermore, 14 participants received booster vaccination with BNT162b2 (n=13) and mRNA-1273 (n=1) 4-6 months after the 2^nd^ vaccination. The amounts of BNT162b2 and mRNA-1273 used for prime doses are 30 μg and 100 μg and for the booster 30 μg and 50 μg respectively. One participant was previously infected with SARS-CoV-2 and subsequently vaccinated with two doses of BNT162b2. The rest of the participants had no SARS-CoV-2 infection prior to vaccination, which was confirmed by testing immunoglobulins (Ig) against N protein (<1.0 COI) and RBD/S1 protein (<0.8 U/ml). Prior to the first vaccination, an assessment regarding a previous infection was possible *via* the presence of anti-N- or/and anti-RBS-Ig. However, after vaccination, a vaccine breakthrough was verified at humoral level solely by the presence of anti-N-antibodies since they were unaffected by vaccination agents. During the study, 3 participants had a breakthrough SARS-CoV-2 infection defined by a positive PCR test on days 83-157 after two vaccinations with BNT162b2. All infected individuals had mild symptoms. Side effects after vaccination were classified as mild or moderate.

### Restoration of diminished vaccine-induced antibody levels after the booster dose

We analyzed the concentrations of total Ig against RBD/S1 protein at different time points (**Figure 1A**) and found its significant elevation after the 1^st^ and 2^nd^ vaccination followed by a substantial decrease before the 3^rd^ vaccination (4-6 months after the 2^nd^ vaccination; p< 0.01; **Figure 1B**). Importantly, the levels of antibodies reached the highest values 2 weeks after the 3^rd^ immunization dose and then significantly decreased after 12 weeks, although remaining higher than those before the 3^rd^ vaccination.

Next, we investigated the influence of vaccine type on the level of antibody production. Homologous mRNA/mRNA vaccination induced significantly higher anti-RBD/S1 Ig production compared to homologous ChAd/ChAd vaccination (**Figure 1C**). Three months after vaccination, participants who received mRNA/mRNA or ChAd/mRNA vaccination showed significantly higher Ig levels than those vaccinated with homologous ChAd/ChAd. Moreover, 3 doses of mRNA vaccines induced a considerably stronger antibody response than two doses of ChAd followed by mRNA vaccination (**Figure 1C**). Interestingly, participants older than 60 years displayed a tendency for decreased values of anti-RBD/S1 Ig after 2^nd^ dose of vaccination as compared to those below 60 (**Figure 1D**).

### Stimulation of SARS-CoV-2-specific CD8 T cells following the booster dose

**Figure 2:**
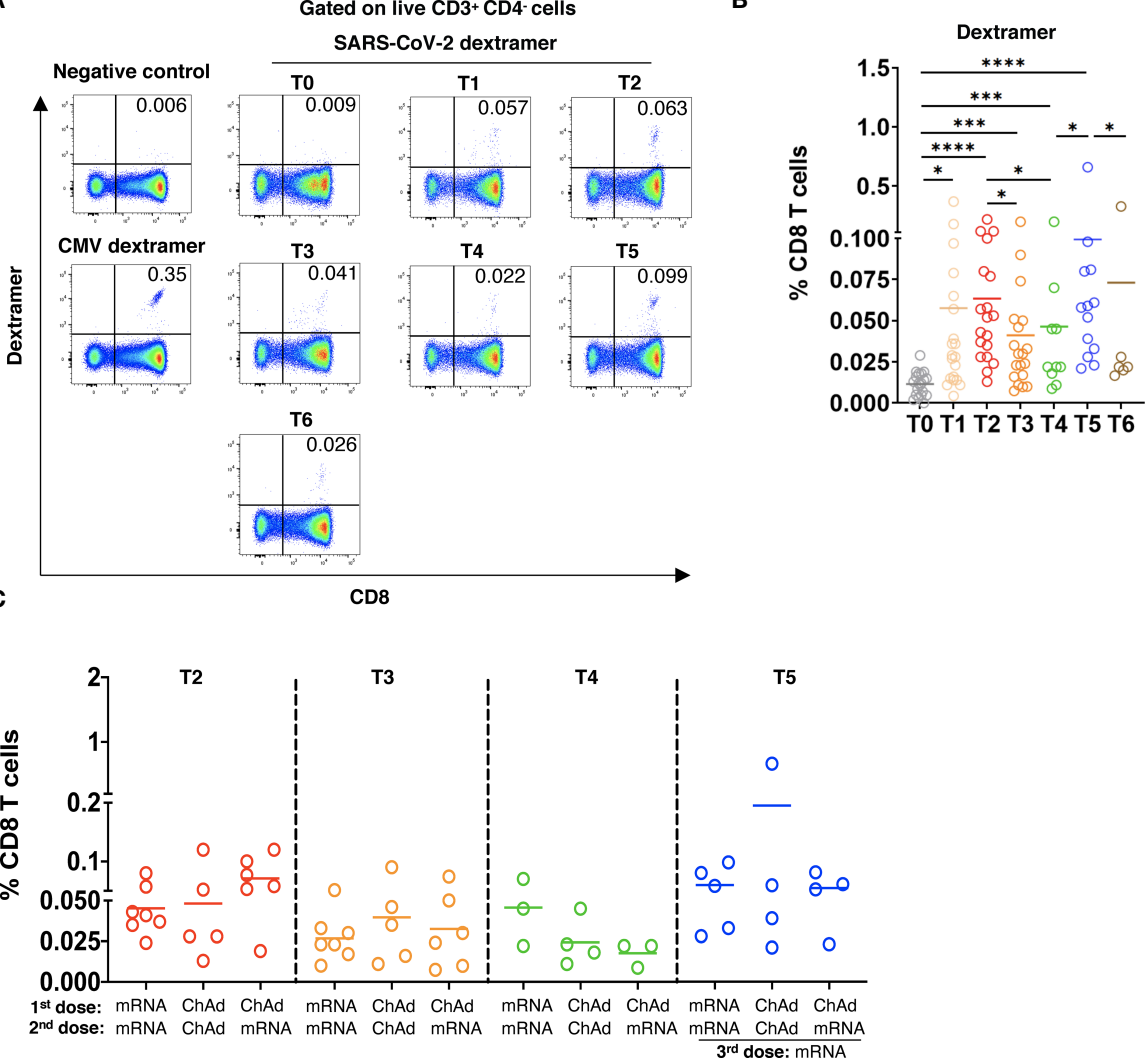
Induction of SARS-CoV-2 specific CD8 T cells after vaccination. PBMC (2x10^6^) were stained with MHC I dextramers containing SARS-CoV-2 spike peptides and the HLA-A allele that was chosen according to the HLA-A typing of each participant (**Supplementary Tables 2, 3**) followed by flow cytometry. **(A)** Representative dot plots for SARS-CoV-2-specific CD8 T cells (dextramer^+^CD8^+^), for cells stained with negative control dextramers (T5), or for CMV specific CD8 T cells (positive control, T5). **(B)** Spike specific CD8 T cells in vaccinated participants are shown as the percentage of dextramer^+^CD8^+^ T cells within total CD8 T cells with the subtraction of positively stained cells in the negative control (n=21). **(C)** Virus specific CD8 T cells in participants vaccinated two times with mRNA (mRNA/mRNA, n=7), two times with ChAd (ChAd/ChAd, n=5) or with ChAd/mRNA (n=5) are shown as the percentage of dextramer^+^CD8^+^ T cells among total CD8 T cells. *P<0.05, ***P<0.001, ****P<0.0001.

To study SARS-CoV-2 spike-specific CD8 T cells, we applied MHC I dextramers containing SARS-Cov-2 spike peptides and the HLA-A allele that was chosen according to the HLA-A typing of each participant (**Supplementary Tables 2, 3**). For dextramer staining, we chose only one HLA-A allele of each participant. Gating strategy for SARS-CoV-2-specific CD8 T cells is shown in **Supplementary Figure 1**. In 3 of 21 participants, we observed dextramer-binding SARS-CoV-2-specific CD8 T cells already at the pre-vaccination time point (T0; **Figure 2A, B**). The frequency of virus-specific CD8 T cells was remarkably elevated after the 1^st^ and 2^nd^ vaccinations. Similar to the antibody levels, the frequency of specific CD8 T cells gradually decreased 12 weeks and 4-6 months after the 2^nd^ dose. The booster dose strongly increased the frequency of SARS-CoV-2-specific CD8 T cells followed by its reduction 3 months after the 3^rd^ vaccination (**Figure 2A, B**). Investigating the effect of the vaccine type on the production of SARS-CoV-2-specific CD8 T cells, we found no significant differences in their frequencies in participants immunized with different vaccines (**Figure 2C**).

**Figure 3:**
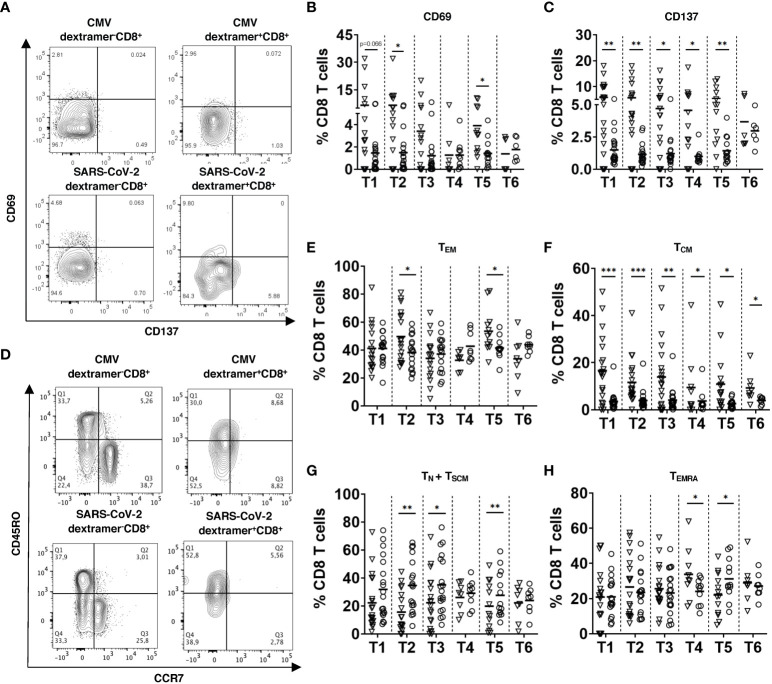
Activation status and memory phenotype of SARS-CoV-2 specific CD8 T cells. PBMC (2x10^6^) were stained with respective SARS-CoV-2 spike or CMV MHC I dextramers and mAbs for CD3, CD4, CD8, CD69, CD137, CD45RO, and CCR7 followed by FACS analysis. **(A)** Representative dot plots for CD69 and CD137 expression on CMV or SARS-CoV-2 dextramer^+^CD8^+^ and dextramer^-^CD8^+^ T cells 2 weeks after the 3^rd^ dose (T5). CD69 **(B)** and CD137 **(C)** expression on SARS-CoV-2 dextramer^+^CD8^+^ and dextramer^-^CD8^+^ T cells from vaccinated individuals (n=19) is presented as the frequency of CD69^+^ or CD137^+^ cells within the CD8 T cell subset. **(D)** Representative dot plots for CD45RO and CCR7 expression on dextramer^+^CD8^+^ and dextramer^-^CD8^+^ T cells at T5. **(E-H)** Results for CD45RO^+^CCR7^-^ effector memory (T_EM_, E), CD45RO^+^CCR7^+^ central memory (T_CM_, F), CD45RO^-^CCR7^+^ naive and stem cell memory (T_N_ + T_SCM_, G) and CD45RO^-^CCR7^-^ terminally differentiated memory T cells (T_EMRA_, H) from vaccinated participants (n=19) are shown as the percentage of corresponding subsets among dextramer^+^CD8^+^ or dextramer^-^CD8^+^ T cells. Data for dextramer^+^CD8^+^ and dextramer^-^CD8^+^ T cells are shown as inverted triangles and circles respectively. Data were excluded when event count <20 in Dextramer^+^ CD8^+^ cells. *P<0.05, **P<0.01, ***P<0.001.

Next, we analyzed the activation status and memory phenotype of induced virus-specific CD8 T cells. Already 2 weeks after 2^nd^ vaccination (T2), dextramer^+^ cells expressed a significantly higher level of CD69 and CD137 as compared to their control counterparts (dextramer^-^ cells), indicating their activation (**Figures 3A-C**). Importantly, the expression of CD69 and CD137 of spike-specific CD8 T cells stayed at the high level over the whole study. Interestingly, we did not see any significant upregulation in the frequency of CD69^+^CD137^+^ subset (data not shown).

Measuring effector memory CD45RO^+^CCR7^-^ SARS-CoV-2-specific CD8 T cells (T_EM_), we demonstrated their increase as compared to their dextramer^-^ counterparts after the 2^nd^ dose of vaccination (T2) followed by a gradual decrease over time and again, a significant elevation after the 3^rd^ dose (T5; **Figures 3D, E**). However, we observed no significant difference between the frequency of T_EM_ between dextramer^+^ and dextramer^-^ cells 3 months after the 3^rd^ dose (T6; **Figures 3D, E**). Interestingly, T_EM_ was found to be a predominant T cell subset among SARS-CoV-2-specific CD8 T cells after the 2^nd^ and 3^rd^ vaccination doses (T2 and T5; **Supplementary Figure 2**). In contrast to T_EM,_ the frequency of CD45RO^+^CCR7^+^ central memory (T_CM_) subset was significantly higher among SARS-CoV-2-specific CD8 T cells than in their control counterparts throughout the vaccination procedure (T1-T6; **Figures 3D, F**). The frequency of CD45RO^-^CCR7^+^ naive and stem memory cells (T_N_+T_SCM_) was significantly lower among virus-specific CD8 T cells than within their dextramer^-^ counterparts (**Figure 3G**). Furthermore, the frequency of terminally differentiated memory T cells (T_EMRA_) among SARS-CoV-2-specific CD8 T cells was higher than in dextramer^-^ cells and remained the largest subset 12 weeks and 4-6 months after the 2^nd^ vaccination (T3 and T4; **Figure 3H** and **Supplementary Figure 2**).

### Induction of functional SARS-CoV-2-specific T cells upon booster vaccination with reactivity to Omicron variant

**Figure 4:**
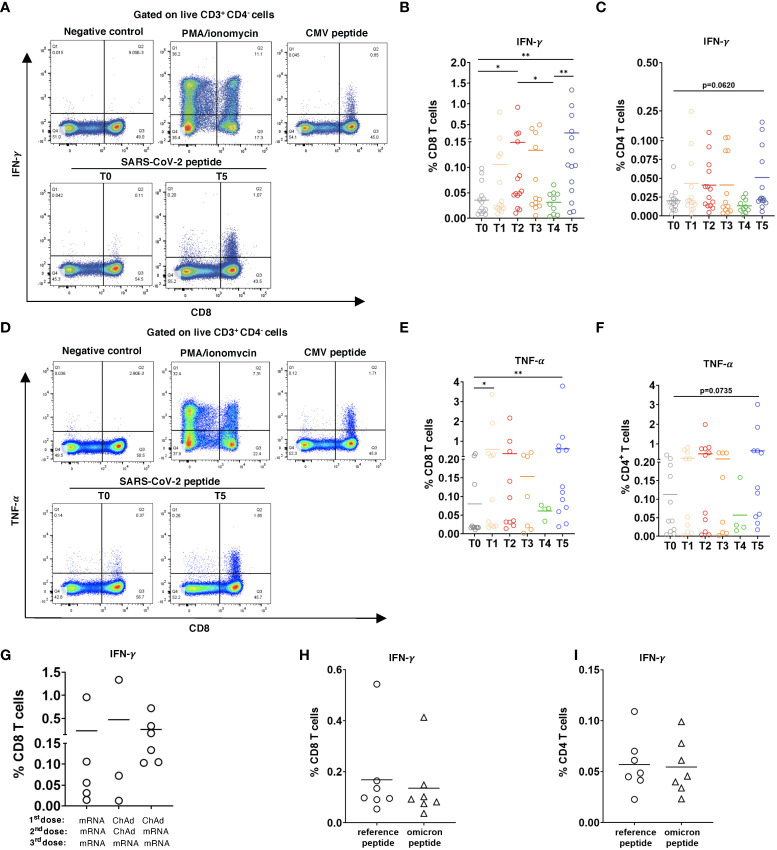
CD8 and CD4 T cell responses to SARS-CoV-2 peptides. PBMC (2x10^6^) were stimulated with SARS-CoV-2 S overlapping peptide pool and anti-CD28 mAb for 6h. The treatment with PMA/ionomycin or CMV peptide pool were used as positive controls, whereas the incubation in medium containing 2% sterile water instead of peptides was considered as negative control. **(A)** Representative dot plots for IFN-γ. production by CD8 T cells at T0 and T5. Negative and positive controls are shown at T5. **(B)** Data are presented as the percentage of IFN-γ^+^ CD8 T cells among total CD8 T cells, **(C)** as well as IFN-γ^+^CD4^+^ T cells among total CD4 T cells with the subtraction of positively stained cells in the negative control (n=14). **(D)** Representative dot plots for TNF-α production by CD8 T cells at T0 and T5. **(D-F)** Results are shown as the percentage of TNF-α^+^ CD8^+^ T cells among total CD8 T cells (n= 11, [**(E)**] and TNF-α^+^CD4^+^ T cells [n=7, **(F)**] among total CD4 T cells. **(G)** Data on IFN-γ producing CD8 T cells in participants vaccinated two times with mRNA (mRNA/mRNA, n=5), two times with ChAd (ChAd/ChAd, n=3) or with ChAd/mRNA (n=6) are presented as the frequency of IFN-γ^+^CD8^+^ T cells among total CD8 T cells. **(H, I)** PBMC were stimulated with Omicron peptide pool and reference peptide pool for 6h in the presence of anti-CD28 mAb. Frequency of IFN-γ^+^CD8^+^ T cells among total CD8 T cells [n=7, **(H)**] and IFN-γ^+^CD4^+^ T cells among total CD4 T cells [n=7, **(i)**] are shown. *P < 0.05, **P < 0.01.

Next, we tested if induced SARS-CoV-2-specific CD8 and CD4 T cells are functionally active based on IFN-γ and TNF-α production. For this, PBMC from 14 participants were stimulated with pooled overlapping 15mer peptides of SARS-Cov 2 Prot_S. Gating strategy for CD8 and CD4 T cells producing IFN-γ and TNF-α is shown in **Supplementary Figure 3**. In 6 of 14 participants, we observed the production of IFN-γ and TNF-α already at the baseline (T0; **Figures 4A, B, E**). In response to peptide stimulation, CD8 T cells collected 2 weeks after the 2^nd^ dose significantly increased IFN-γ production (T2; **Figure 4B**). Similar to other tested immune parameters, the level of IFN-γ was reduced 4-6 months after the 2^nd^ vaccination and was remarkably enhanced following the 3^rd^ dose (T4 and T5 respectively; **Figure 4B**). Similar to IFN-γ CD8 T cells produced also significantly higher amounts of TNF-α after the 3^rd^ dose (**Figures 4D, E**). Furthermore, we found that CD4 T cells could also increase the secretion of IFN-γ and TNF-α after 3^rd^ vaccination in response to peptide stimulation, although to a lesser extent than CD8 T cells (**Figure 4C, F**). In addition, no significant effect of the type of vaccine was found on the capacity of CD8 T cells to increase the production of IFN-γ (**Figures 4G**) and TNF-α (data not shown). To assess the T cell response of vaccinated participants against the Omicron variant, we measured IFN-γ production by CD8 and CD4 T cells taken from 7 participants 2 weeks after the 3^rd^ dose and stimulated with pooled overlapping 15mer peptides spanning the mutated region in Omicron spike protein. The reference peptide pool covering the homologous domains of the Wuhan variant was used as a control. Although we observed a tendency of decreased level of IFN-γ response against the Omicron peptides by both CD8 and CD4 T cells as compared to the reference peptides, the difference was not statistically significant (**Figures 4H, I**).

### Infection after vaccination promotes a higher level of T cells than the booster dose

**Figure 5:**
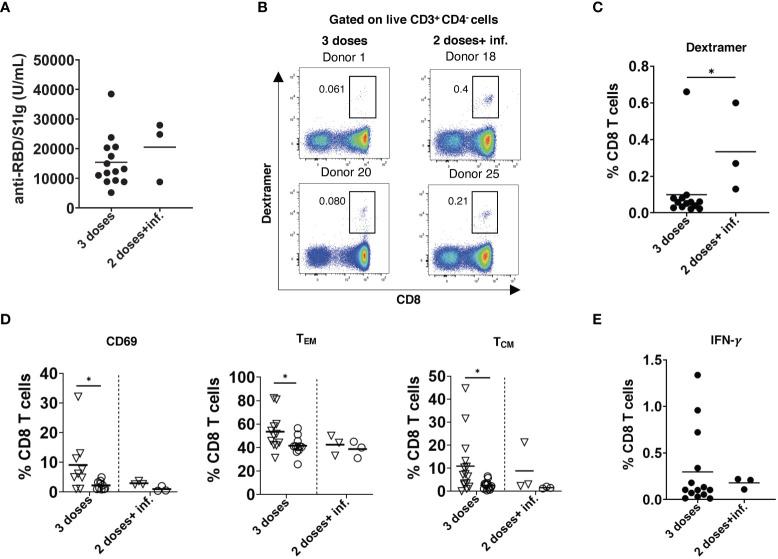
Effect of breakthrough SARS-CoV-2 infection on antibody and T cell responses in vaccinated participants. **(A)** Total RBD/S1 Ig were measured in the serum of participants infected after 2 doses of vaccination (2 doses + inf.; n=3) or after 3^rd^ dose (n=14) and expressed as U/mL. **(B)** Representative dot plots for SARS-CoV-2 spike specific CD8 T cells (dextramer^+^CD8^+^) from both groups. **(C)** Spike-specific CD8^+^ T cells infected after 2 doses of vaccination (n=3) or after 3^rd^ dose (n=14) are shown as the percentage of dextramer^+^CD8^+^ T cells within total CD8 T cells. **(D)** Frequency of spike-specific CD69^+^, T_EM_, and T_CM_ CD8 T cells after infection (n=3) and 3^rd^ dose of vaccination (n=14) are presented as the percentage within total CD8 T cells. Dextramer^+^CD8^+^ and dextramer^-^CD8^+^ are indicated as inverted triangles and circles, respectively. **(E)** PBMC were stimulated with SARS-CoV-2 S peptide pool and anti-CD28 mAb for 6h. Data are presented as the percentage of IFN-γ^+^CD8^+^ T cells among total CD8 T cells (n=3 and 14 respectively). *P < 0.05.

Breakthrough infections after the 2^nd^ dose of vaccination occurred in three participants approximately 4 months after the 2^nd^ dose. The level of anti-RBD/S1 antibodies measured around 3 weeks after the disease recovery was only slightly higher than 2 weeks after the 3^rd^ dose of vaccination (T5) in non-infected participants (**Figure 5A**). On the contrary, breakthrough infections induced significantly elevated frequencies of SARS-CoV-2-specific CD8 T cells as compared to those after the 3^rd^ dose (**Figures 5B, C**). However, these CD8 T cells showed no upregulation of CD69 and accumulation of cells with memory phenotype, in contrast to CD8 T cells measured after the 3^rd^ dose (**Figure 5D**). In addition, we observed no differences in the frequency of IFN-γ producing CD8 T cells stimulated by breakthrough infections or the 3^rd^ vaccination dose (**Figure 5E**).

In one participant infected with SARS-CoV-2 before vaccination, we found an increased level of antibodies after the 2^nd^ dose as compared to that in uninfected participants after the 3^rd^ dose, whereas the frequency of SARS-COV-2-specific CD8 T cells was lower at this time point (**Supplementary Table 4**).

### Vaccination promoted upregulation of PD-L1 expression on CD14^+^HLA-DR^+^ monocytes

**Figure 6:**
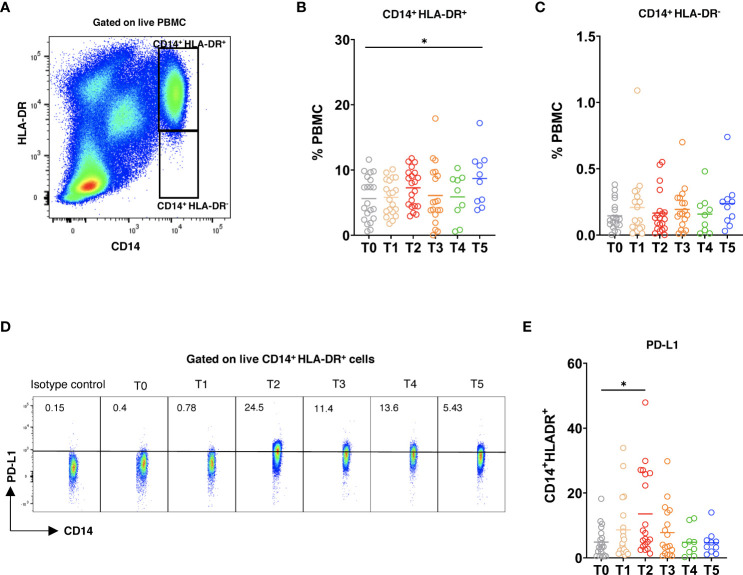
Myeloid cell phenotype after vaccination. PBMC were stained with 7AAD, CD14, HLA-DR, and PD-L1 mAbs and measured by flow cytometry. **(A)** Representative dot plots for CD14^+^HLA-DR^+^ and CD14^+^HLA-DR^-^7AAD^-^ live myeloid cells. CD14^+^HLA-DR^+^ **(B)** and CD14^+^HLA-DR^-^ **(C)** cells were analyzed at different time points and are shown as the frequency of respective subset among total PBMC (n=21). **(D)** Representative dot plots for PD-L1 expression on CD14^+^HLA-DR^+^ cells. **(E)** Data are presented as the percentage of PD-L1^+^CD14^+^HLA-DR^+^ cells among total CD14^+^HLA-DR^+^ cells. *P < 0.05.

Finally, we studied the alterations of circulating myeloid cells following the vaccination. After the 3^rd^ dose (T5), the frequency of CD14^+^HLA-DR^+^ cells was elevated as compared to the baseline (**Figures 6A, B**). Moreover, the frequency of CD14^+^HLA-DR^-^ myeloid cells within PBMC showed a slight increase at this time point (**Figure 6C**). Interestingly, the expression of PD-L1 was significantly upregulated on CD14^+^HLA-DR^+^ monocytes after the 2^nd^ dose (**Figures 6D, E**), whereas the frequency of PD-L1^+^ CD14^+^HLA-DR^-^ cells remained low at different time points after vaccination (**Supplementary Figure 4A**). In addition, we observed no statistically significant differences in the production of reactive oxygen species (ROS) and nitric oxide (NO) by both CD14^+^HLA-DR^+^ and CD14^+^HLA-DR^-^ myeloid cells upon the vaccination (**Supplementary Figures 4B-E**).

## Discussion

In this study, we observed a gradual decrease in anti-RBD/S Ig concentrations in the serum of participants within 6 months after the 2^nd^ vaccination dose that was in agreement with a previous publication, demonstrating a substantial decrease of anti-S neutralizing Ig after the 2^nd^ dose of BNT162b2 (31). Moreover, it was reported that aged participants displayed lower antibody level and its more drastic decrease over time after vaccination (32). Here, we also observed lower and less persistent antibody levels in participants >60 years old than those in younger donors. However, these differences were not statistically significant since the antibody response was heterogeneous due to the different vaccination settings applied in this study. In addition, each dose of mRNA-1273 contains more mRNA than BNT162b2, which might explain the heterogeneity in antibody responses.

Analyzing the effect of different vaccines, we demonstrated that the injection of ChAd followed by mRNA vaccines or homologous mRNA/mRNA setting induced a higher level of anti-RBD/S1 Ig than ChAd/ChAd immunization. These data are in accordance with that from Barros-Martins et al. (33) who reported stronger antibody responses against SARS-CoV-2 after heterologous mRNA/ChAd vaccination. Our findings showed that booster mRNA dose elevated anti-RBD/S1 Ig levels, which waned after 6 months. Other authors also observed an augmentation of humoral immune responses induced by the booster dose using BNT162b2 (34). Therefore, the administration of booster doses seems to be critically important for the generation of anti-spike SARS-CoV-2 antibodies.

In parallel to augmented antibody responses, we observed a strong induction of virus-specific CD8 T cells (measured by dextramer staining) followed by a substantial reduction 4-6 months after the 2^nd^ dose and reaching a maximum after the 3^rd^ vaccination that was also reported by others (35, 36). Intriguingly, in contrast to humoral responses, we detected no differences in the induction of anti-viral CD8 T cells in various vaccination settings.

It is known that long-lived memory T cells are required to protect the host from re-infection. Consistent with the recent publications (31, 37), we found that SARS-CoV-2-specific memory CD8 T cells peaked 2 weeks after the 2^nd^ dose and remained detectable for 4-6 months. Importantly, the boost vaccination generated a robust expansion of effector and central memory CD8 T cells. Furthermore, we found that SARS-CoV-2-specific CD8 T cells had predominantly a T_EMRA_ phenotype after 4-6 months that is in accordance with another report showing that T_EM_ emerged after SARS-CoV-2 infection converted over time to T_EMRA_ phenotype (38). Additionally, virus-specific CD8 T_EMRA_ cells were found to be dominant in convalescent COVID-19 individuals (39). Since these cells represent a heterogeneous population (40), more studies are required to decipher their importance for long-lasting anti-viral immunity.

We also found that virus-specific CD8 T cells upregulate the activation markers CD69 and CD137. Although some previous papers reported that most of these cells expressed both markers (41, 42), we failed to detect any double positive SARS-CoV-2-specific CD8 T cells, indicating that different T cell subsets express CD69 or CD137. Moreover, we tested if spike-specific T cells were functionally active by measuring intracellular IFN-γ and TNF-α in CD8 and CD4 T cells upon the stimulation with pooled overlapping 15mer peptides. Functional CD8 T cell responses were markedly increased after the 2^nd^ with a maximum after the 3^rd^ vaccination, whereas the stimulation of cytokine production by CD4 T cells was lower. In line with this, it was previously demonstrated that the functionality of SARS-CoV-2-specific CD4 T cells induced by vaccination was reduced compared to that of CD8 T cells (43). Intriguingly, in contrast to humoral responses, we detected no differences in the cytokine production by CD8 T cells upon viral peptide stimulation in various vaccination settings.

In some donors, we found SARS-CoV-2-specific T cells before vaccination, although they have no detectable Ig against nucleocapsid protein, which excludes the possibility of pre-exposure to SARS-CoV-2. Similarly, previous publications described SARS-CoV-2-specific T cells in individuals without the history of COVID-19 (44–46). This might be explained by the pre-exposition of such participants to other coronaviruses that induce T cells cross-reacting to SARS-CoV-2 (47). However, the presence of pre-existing virus-specific CD8 T cells had no effect on the efficiency of functional T cell induction upon vaccination.

Decay in humoral and T cell immune response and against emerging new SARS-CoV-2 variants might lead to breakthrough infections (48). In our study, 3 participants were infected with SARS-CoV-2 approximately 5 months after the 2^nd^ dose of mRNA vaccination. Interestingly, such breakthrough infection induced higher numbers of SARS-CoV-2-specific T cells than the booster vaccination, whereas the antibody response does not significantly differ. Since the Omicron variant has become the dominant one, leading to breakthrough infections, we investigated functional reactivity of T cells from participants receiving the 3^rd^ vaccination dose against this variant. We found no statistically significant differences between IFN-γ production in T cells stimulated by the Omicron and reference peptides. Our findings are in agreement with the studies demonstrating a minimal escape of the Omicron variant from T cell immune response (49, 50). Although it might not be sufficient for preventing infection, an existing CD8 T cell immune response against Omicron might help to reduce disease severity.

Although some reports demonstrated dysregulation of monocytic cells in COVID-19 infection (51, 52), the dynamics of such alterations during vaccination are not clear. Our findings demonstrated that the frequency of CD14^+^HLA-DR^+^ monocytes is enhanced after the booster dose. In line with our findings, Liu et al. (53) reported that the frequency of CD14 monocytes was increased following vaccination. Moreover, it was demonstrated that different monocyte subsets were emerged during the disease progression in COVID-19 patients (54). Further studies are needed to characterize monocyte changes upon vaccination. Interestingly, we failed to observe the vaccination-induced accumulation of CD14^+^HLA-DR^-^ cells that could be considered as a counterpart of MDSC in donors. In contrast, Falck-Jones et al. (29) and Sacchi et al. (55) reported an expansion of MDSC that inhibit T cell responses in patients with severe COVID-19. This difference could be due to a long-term virus persistence in COVID-19 patients supporting the accumulation of MDSC. The upregulation of PD-L1 expression as well as the production of ROS and NO are important strategies used by MDSC to suppress T cell responses (27). Therefore, we examined these parameters in both myeloid subsets and found that PD-L1 expression is upregulated on CD14^+^HLA-DR^+^ monocytes after the 2^nd^ dose, which could be characterized as a protective mechanism suppressing overactivated T cells induced by vaccines.

Taken together, our data demonstrated that the 3^rd^ (booster) dose of vaccination induced a maximum production of anti-RBD/S1 Ig and functionally active SARS-CoV-2-specific CD8 T cells, showing memory phenotype and secreting the cytokines IFN-γ and TNF-α. Importantly, we found no accumulation of myeloid cells with MDSC-like immunosuppressive phenotype following the vaccination. An observed decrease in both antibody and T cell responses 3 months after the booster dose could lead to breakthrough infections in some cases, indicating that further booster vaccinations should be implemented to achieve long-term immunity against COVID-19 caused by SARS-CoV-2.

## Data availability statement

The original contributions presented in the study are included in the article/**Supplementary Material**. Further inquiries can be directed to the corresponding author.

## Ethics statement

This study was reviewed and approved by the Ethics Committee of University Medical Center Mannheim (2010-318N-MA and 2020-556N). The participants provided their written informed consent to participate in this study.

## Author contributions

VU, JU, and MN designed the study. FK, AL, SL, IA, and RB performed the experiments. FK performed the data analysis. CGe, MR measured the antibodies by ECLIA. PB contributed to the HLA typing of the donors. CGo contributed to the design and analysis of T cells by flow cytometry. FK and VU wrote the manuscript. All authors contributed to the article and approved the submitted version.

## Funding

This work was supported by the German Academic Exchange Service (DAAD to FK), the German Federal Ministry of Education and Research (BMBF) – SERPENTINE project in the ERA PerMed network (01KU2017 to VU), the German Research Foundation (DFG) – project number 259332240/RTG 2099 (to JU and VU).

## Acknowledgments

We thank Yvonne Nowak, Sayran Arif-Said (both Department of Dermatology, Venereology and Allergology, University Medical Center Mannheim) and Ludmila Umansky (Department of Department of Neurology, University Medical Center Mannheim) for the preparation of PBMC and serum samples, Ameli Götz and Ruth Leiblein (Institute for Clinical Chemistry, Medical Faculty Mannheim, University of Heidelberg) for help with the antibody detection and Frank Stötzer (Institute of Transfusion Medicine and Immunology, Heidelberg University) for HLA typing of the donors and Immudex (Copenhagen, Denmark) for providing MHC I Dextramer^®^ reagents. We thank all donors for their participation.

## Conflict of interest

The authors declare that the research was conducted in the absence of any commercial or financial relationships that could be construed as a potential conflict of interest.

## Publisher’s note

All claims expressed in this article are solely those of the authors and do not necessarily represent those of their affiliated organizations, or those of the publisher, the editors and the reviewers. Any product that may be evaluated in this article, or claim that may be made by its manufacturer, is not guaranteed or endorsed by the publisher.
