# Supplementary material for: Booster dose of mRNA vaccine augments waning T cell and antibody responses against SARS-CoV-2
Source: Front Immunol. 2022 Oct 12;13:1012526. doi: 10.3389/fimmu.2022.1012526 (PMC9597683; doi:10.3389/fimmu.2022.1012526)
Supplement: Supplementary file 1 [file DataSheet_1.docx]

**Supplementary Figure 1.** **Gating strategy for SARS-CoV-2 specific CD8 T cells.** PBMC were stained with SARS-CoV-2 MHC I dextramers corresponding to HLA-A alleles of each participant as well as with mAbs for CD3, CD4, and CD8 to differentiate between virus specific (dextramer^+^) CD8 T cells and dextramer^-^CD8^+^ T cells. Expression of CD45RO, CCR7, CD69, and CD137 were further determined on dextramer^+^CD8 and dextramer^-^CD8^+^ T cells. FMO: Fluorescence minus one.

**Supplementary Figure 2. Distribution of memory T cell subsets among SARS-CoV-2 specific CD8 T cells.** PBMC from vaccinated individuals were stained with spike MHC I dextramers, CD3, CD4, CD8, CD45RO and CCR7 mAbs. Data from Donor 1 are presented as pie charts showing the proportion of T_EM_, T_CM_, T_N_+T_SCM_, and T_EMRA_ cells among dextramer^+^CD8^+^ and dextramer^-^CD8^+^ cells at different time points upon vaccination.

**Supplementary Figure 3. Gating strategy for cytokine producing CD8 and CD4 T cells.** PBMC were stimulated with SARS-CoV-2 peptide pools and stained with mAbs for CD3, CD8, CD4 and IFN-γ (or TNF-α) to detect functionally active cytokine secreting CD8 and CD4 T cells.

**Supplementary Figure 4. Effect of vaccination on immunosuppressive marker expression on myeloid cells.** The expression of PD-L1 and production of reactive oxygen species (ROS) and nitric oxide (NO) was measured by flow cytometry. (A) Data on PD-L1 expression are presented as the percentage of PD-L1^+^CD14^+^HLA-DR^-^ cells among total CD14^+^HLA-DR^-^ population. (B-E), The level of ROS (B, C) and NO (D, E) production by CD14^+^HLA-DR^+^ (B, D) and CD14^+^HLA-DR^-^ cells (C, E) at various time points upon vaccination is shown as median fluorescence intensity (MFI).
