# Supplementary material for: Booster dose of mRNA vaccine augments waning T cell and antibody responses against SARS-CoV-2
Source: Front Immunol. 2022 Oct 12;13:1012526. doi: 10.3389/fimmu.2022.1012526 (PMC9597683; doi:10.3389/fimmu.2022.1012526)
Supplement: Supplementary file 2 [file DataSheet_2.docx]

**Total number of participants (n=25)**

| **Type of vaccine mRNA/ mRNA ChAd/ ChAd**  **(1^st^ dose, 2^nd^ dose), n** 13 6  (BNT162b2, 12 )  (mRNA-1273, 1)    Mean age, yrs (range) 40( 24-74) 52 (29-66)  <60 yrs, n 12 2    $\geq$60 yrs, n 1 4 | **ChAd/ mRNA**  6  39 (29-64)  5  1 | |
| --- | --- | --- |
| Sex, n |  |  |
| Male 7 2 | 1 |  |
| Female 6 4  Type of booster dose  BNT162b2 4 5  mRNA-1273 1  SARS-CoV-2 infection    Pre-vaccination 1    After 2 doses 3 | 5    4 |  |
|  |  |  |

**Supplementary Table 1:** Characteristics of the study participants.

| **Donor ID** | **HLA-A^*^** | **HLA-A^*^** |
| --- | --- | --- |
| D1 | 01:01 | 11:01 |
| D2 | 01:01 | 02:01 |
| D3 | 01:01 | 25 |
| D4 | 01:01 | 24:02 |
| D5 | 01:01 | 02:01 |
| D6 | 01:01 | 30 |
| D7 | 03:01 | 24:02 |
| D8 | 02:01 | 68:01 |
| D9 | 02:01 | 68:02 |
| D10 | 02:01 | 02:05 |
| D11 | 02:01 |  |
| D12 | 02:01 | 31:01 |
| D13 | 02:01 | 24:03 |
| D14 | 02:01 | 24:02 |
| D15 | 02:01 | 24:02 |
| D16 | 02:01 | 24:02 |
| D17 | 02:01 | 31 |
| D18 | 02:01 | 24:02 |
| D19 | 02:01 | 29:02 |
| D20 | 03:01 |  |
| D21 | 03:01 | 26 |
| D22 | 03:01 | 24:02 |
| D23 | 24:02 | 30 |
| D24 | 24:02 | 32 |
| D25 | 01:01 | 11:01 |

**Supplementary Table 2:** HLA-A alleles of participants.

| **Reagent** | **Fluorescence** | **Company** | **Dilution** |
| --- | --- | --- | --- |
| Anti-human CD3 (clone:SK7) | APC-Cy7 | BD Biosciences | 1:100 |
| Anti-human CD4 (clone: SK3) | R718 | BD Biosciences | 1:100 |
| Anti-human CD8 (clone: RPA-T8) | PE-Cy7 | BD Biosciences | 1:100 |
| Anti-human CD45RO (clone: UCHL1) | BB515 | BD Biosciences | 1:100 |
| Anti-human CCR7 (clone: 2-L1-A) | BV605 | BD Biosciences | 1:100 |
| Anti-human CD69 (clone:L78) | APC | BD Biosciences | 1:100 |
| Anti-human CD137/41BB (clone: 4B4-1) | BV421 | BD Biosciences | 1:100 |
| Fixable Viability Stain | BV510 | BD Biosciences | 1:1000 |
| Anti-human IFN-$\gamma$ (clone: B27) | PE | BD Biosciences | 1:25 |
| Anti-human TNF-$\alpha$ (clone: MAb11) | PE | BD Biosciences | 1:25 |
| Anti-human CD14 (clone: MφP9) | APC-Cy7 | BD Biosciences | 1:100 |
| 7AAD Staining Solution | PerCP-Cy5.5 | Miltenyi Biotech | 1:200 |
| Anti-human HLA-DR (clone: G46-6) | V500 | BD Biosciences | 1:100 |
| Anti-human PD-L1 (clone: MIH4) | BV421 | BD Biosciences | 1:100 |
| LTDEMIAQY (SARS-CoV-2 S_255-263_ ) HLA-A*0101 | PE | Immudex | 1:20 |
| WTAGAAAYY (SARS-CoV-2 S_958-966_ ) HLA-A*0101 | PE | Immudex | 1:20 |
| VLNDILSRL (SARS-CoV-2 S_958-966_) HLA-A*0201 | PE | Immudex | 1:20 |
| YLQPRTFLL (SARS-CoV-2 S_269-277_) HLA-A*0201 | PE | Immudex | 1:20 |
| KCYGVSPTK (SARS-CoV-2 S_378-386_) HLA-A*0301 | PE | Immudex | 1:20 |
| GVYFASTEK (SARS-CoV-2 S_89-97_) HLA-A*0301 | PE | Immudex | 1:20 |
| QYIKWPWYI (SARS-CoV-2 S_1205-1213_) HLA-A*2402 | PE | Immudex | 1:20 |
| KWPWYIWLGF (SARS-CoV-2 S_1211-1220_) HLA-A*2402 | PE | Immudex | 1:20 |
| NLVPMVATV (CMV pp65_495-504_ ) HLA-A*0201 | PE | Immudex | 1:20 |
| ALIAPVHAV HLA-A*0201 (negative control) | PE | Immudex | 1:20 |

**Supplementary Table 3:** Reagents used in flow cytometry.

|  | **Time of blood sampling** | **Anti-S Ig levels (U/mL)**  **mean**$\boldsymbol{\pm}$**SD** | **Anti-N Ig levels (U/mL)**  **mean**$\boldsymbol{\pm}$**SD** | **Dextramer^+^CD8^+^ T cells (% of PBMC)**  **mean**$\boldsymbol{\pm}$**SD** |
| --- | --- | --- | --- | --- |
| **Infected pre-vaccination, (Donor 17)** | 8 months after infection (pre-vaccination) | 92.4 | 29.3 | 0.023 |
|  | 3 weeks after 1^st^ dose | 4902 | 35.7 | 0.029 |
|  | 12 weeks after 1^st^ dose | 10932 | 24 | 0.026 |
|  | Before 2^nd^ dose | 5008 | 18.3 | 0.025 |
|  | 3 weeks after 2^nd^ dose | 24722 | 18.7 | 0.035 |
| **Infection-naive donors**  **(n=21)** | Pre-vaccination | 0 | 0 | 0.01$\pm$0.009 |
|  | 2-3 weeks after 1^st^ dose | 2414$\pm$10689 | 0 | 0.056$\pm$0.048 |
|  | 2 weeks after 2^nd^ dose | 7863$\pm$5228 | 0 | 0.06$\pm$0.086 |
|  | 12 weeks after 2^nd^ dose | 2444$\pm$1456 | 0 | 0.049$\pm$0.044 |
|  | Before 3^rd^ dose | 1586$\pm$2607 | 0 | 0.035$\pm$0.019 |
|  | 2 weeks after 3^rd^ dose | 15423$\pm$8467 | 0 | 0.21$\pm$0.48 |

**Supplementary Table 4:** Comparison of antibody levels and frequency of SARS-CoV-2 specific CD8^+^ T cells between pre-infected donor (Donor 17) and uninfected donors.
